# RETRACTED ARTICLE: Pulmonary Isolation of Multidrug resistant “*Mycobacterium simulans*” and *Mycobacterium tuberculosis* from a patient in the Horn of Africa

**DOI:** 10.1038/s41598-018-33737-9

**Published:** 2018-10-26

**Authors:** Fériel Bouzid, Djaltou Aboubaker Osman, Emeline Baptiste, Jeremy Delerce, Mohamed Osman Hassan, Warsama Ibrahim Arreh, Anthony Levasseur, Eric Garnotel, Michel Drancourt

**Affiliations:** 1 Aix Marseille Univ, IRD, MEPHI, IHU Méditerranée Infection, Marseille, France; 2 Centre d’Études et de Recherche de Djibouti (CERD), Institut de Recherche Médicinale (IRM), Djibouti, Djibouti; 3 Hôpital d’instruction des armées Alphonse Laveran, Marseille, France; 4 Hôpital pneumo-phtisiologie Chakib Saad Omar, Djibouti, Djibouti

Retraction of: *Scientific Reports* 10.1038/s41598-018-33737-9, published online 26 October 2018

Editors have retracted this Article.

After publication of this paper concerns about ethical oversight of this study were brought to the attention of the Editors. The paper cites approval from an institutional ethics committee in France, but the sample used in this study was sourced from a patient in Djibouti. The Authors were not able to provide documentation of approval from an ethics committee in this country, or of compliance with local regulations regarding the use of such samples in research. The Authors were also not able to provide documentation demonstrating that consent from the patient to publish this case report was secured. The content of this article has therefore been removed to protect patient confidentiality.

Fériel Bouzid and Djaltou Aboubaker Osman disagree with this retraction. Jeremy Delerce, Mohamed Osman Hassan, Warsama Ibrahim Arreh, Anthony Levasseur, & Eric Garnotel did not respond to the correspondence from the Editors about this retraction. Michel Drancourt did not explicitly state if he agrees or disagrees with the retraction. The Editors were not able to obtain the current contact details of Emeline Baptiste.

